# Stem-Cell-Regenerative and Protective Effects of Squid (*Symplectoteuthis oualaniensis*) Skin Collagen Peptides against H_2_O_2_-Induced Fibroblast Injury

**DOI:** 10.3390/md22060255

**Published:** 2024-05-30

**Authors:** Mingjun Wei, Lakshmi Jeevithan, Na Li, Lixin Liu, Jiren Xu, Wenhui Wu, Jeevithan Elango

**Affiliations:** 1 Department of Marine Biopharmacology, College of Food Science and Technology, Shanghai Ocean University, Shanghai 201306, China; weimj98@163.com (M.W.); lakshmijeevithan@gmail.com (L.J.); 18335440803@163.com (N.L.); 18651125617@163.com (L.L.); mikumiyo@hotmail.com (J.X.); 2 Putuo Sub-Center of International Joint Research Center for Marine Biological Sciences, Zhongke Road, Putuo District, Zhoushan 316104, China; 3 Center of Molecular Medicine and Diagnostics (COMManD), Department of Biochemistry, Saveetha Dental College and Hospitals, Saveetha Institute of Medical and Technical Sciences, Saveetha University, Chennai 600077, India; 4 Department of Biomaterials Engineering, Faculty of Health Sciences, UCAM Universidad Católica San Antonio de Murcia, 30107 Murcia, Spain

**Keywords:** squid collagen peptides, skin cells, H_2_O_2_ oxidative damage, regenerative medicine

## Abstract

Recently, there has been a growing interest in collagen peptides derived from marine sources for their notable ability to protect skin cells against apoptosis induced by oxidants. Therefore, the current study aimed to investigate the fundamental properties of collagen peptides, including their physicochemical, thermal, structural, stem-cell-regenerative, and skin-cell-protective effects, in comparison to commercial collagen peptides. The acid-soluble (ASC) and pepsin-soluble (PSC) collagens exhibited three distinct bands on SDS-PAGE, namely α (α_1_ and α_2_), β, and γ chains, confirming a type I pattern. The thermal profiles obtained from TG and DSC analyses confirmed the denaturation of PSC and ASC at temperatures ranging from 51.94 to 56.4 °C and from 52.07 to 56.53 °C, respectively. The purified collagen peptides were analyzed using SDS-PAGE and MALDI-TOF mass spectrometry, revealing a mass range of 900–15,000 Da. Furthermore, the de novo peptide sequence analysis confirmed the presence of the Gly-X-Y repeating sequence in collagen peptides. Collagen peptide treatments significantly enhanced HFF-1 cell proliferation and migration compared to the control group. ELISA results confirmed the potential interactions between collagen peptides and HFF-1 cells through α_2_β_1_, α_10_β_1_, and α_11_β_1_ integrin receptors. Notably, collagen peptide treatment effectively restored the proliferation of HFF-1 cells damaged by H_2_O_2_. Consequently, the advantageous characteristics of squid skin collagen peptides highlight their promising role in regenerative medicine.

## 1. Introduction

Collagen, the primary extracellular matrix protein of animal connective tissue, plays a crucial role in maintaining skin elasticity, firmness, and tissue support. Its unique helical structure consists of three polypeptide chains with Gly-X-Y tripeptide sequences, where X and Y are typically proline and hydroxyproline residues. In 2018, the global collagen market size was estimated at approximately USD 4.27 billion [1]. The increasing demand for collagen in various sectors such as dental surgeries, tissue engineering, and bone grafting, as well as in the food and pharmaceutical industries, has led to a rise in collagen extraction. As a result, the global collagen market size has grown significantly and is projected to reach around USD 6.63 billion by 2025 [2]. Recent studies have shown that collagen peptides exhibit superior biological properties such as anti-cancer, wound-healing, anti-proliferative, and anti-aging effects compared to native collagen [3,4,5].

Recently, there has been an increasing interest in peptides obtained from the enzymatic degradation of marine organisms because of their ability to combat oxidative stress, delay the aging process, and promote wound healing. Various marine resources like fish, squid, octopus, seahorse, sea cucumber, shark, and whale have been utilized to extract bioactive peptides for different medical purposes [6,7]. Squid, in particular, stands out as a highly sought-after seafood and a valuable protein source among the different marine raw materials available.

The collagenous remnants (found in both the inner and outer tunics) produced during the processing of the squid *Symplectoteuthis oualaniensis* are a significant source of bioactive peptides [8,9]. This particular squid species thrives in a dark deep-sea habitat characterized by high pressure, low temperature, and low oxygen levels. As a result, it displays excellent growth attributes, including a short growth cycle and rapid reproduction rate, which enhances its potential for producing novel bioactive compounds. Collagens have been extracted from the skins of various squid species such as *Doryteuthis singhalensis*, *Dosidicus gigas*, and *Loligo formosana* [10,11,12]. 

The proteins obtained from squid (*Symplectoteuthis oualaniensis*) were subjected to hydrolysis using protease at a designated cleavage site. This process resulted in the production of small molecular peptides and free amino acids, which exhibited high absorption activity [13]. Extensive research has confirmed that these proteins possess specific antioxidant activity [14,15], hypoglycemic activity [16,17], and various other biological activities. As a result, they find extensive applications in the cosmetic and pharmaceutical industries. Several studies reported the antioxidant, antimutagenic, antiproliferative, anti-hyaluronidase, anti-tyrosinase, ACE-inhibitory, and anti-cancer activities of squid skin collagen hydrolysates/peptides [18,19,20,21].

Our previous research showed the effective extraction of collagen hydrolysates from squid and verified their ability to protect against H_2_O_2_ tissue damage as antioxidants [9]. These findings provide strong evidence that the protein hydrolysate derived from *S. oualaniensis* squid is a remarkable source for obtaining antioxidant peptides. Nevertheless, there is currently no research on the potential impact of squid (*S. oualaniensis*) collagen peptides on oxidative protection. Therefore, this study aimed to extract collagen peptides from squid skin collagen and evaluate their biochemical, structural, thermal, and rheological characteristics in terms of their abilities to regenerate stem cells and provide oxidative protection.

## 2. Results and Discussion

### 2.1. Electrophoretic Pattern

**Figure 1:**
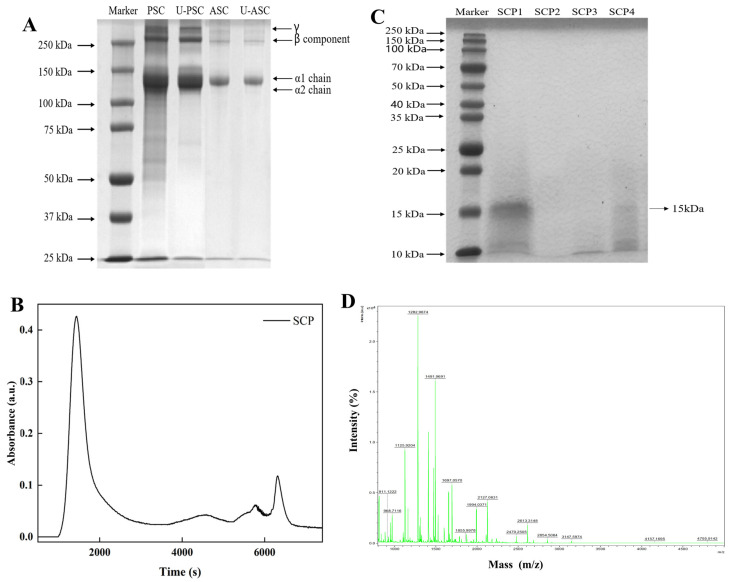
SDS-PAGE patterns (**A**) of PSC and ASC from squid skin on 7.5% gel. Lane 1, molecular weight standard marker; lane 2, PSC; lane 3, U-PSC; lane 4, ASC; lane 5, U-ASC. The elution curve of Sephadex G-50 (**B**) and SDS-PAGE protein pattern (**C**) of SCP on 12.5% gel. Lane 1, molecular weight standard marker; lane 2–5, four components collected after elution. MALDI-TOF mass spectrometry (**D**) of SCP; obtained mass range: 900–5000 amu.

Electrophoretic protein profiles (Figure 1A) revealed the molecular masses of PSC and ASC extracted from squid skin. The gel analysis demonstrated two distinct bands within the range of 100 kDa to 150 kDa, namely α_1_ (132 kDa; 130 kDa) and α_2_ (128 kDa; 120 kDa) chains, respectively. The electrophoretic pattern revealed that the collagen extracted from *S. oualaniensis* skin exhibited a type I configuration, characterized by [α_1_(I)]_2_α_2_ arrangement. However, the molecular weight of α_2_ chain in PSC was determined to be similar to that of the α_1_ chain. Moreover, higher-molecular-weight bands were observed in the range exceeding 180 kDa, comprising a quantity of γ chains (trimeric form of α chains) and β chains (dimeric form of α chains), with a relatively higher proportion of β chains, thereby indicating extensive cross-linking within the collagen structure [22]. The findings indicated that the squid skin-derived collagen displayed the usual traits of type I collagen, with similar protein profiles found in various other species of squid [23,24,25,26]. The darker protein bands of PSC compared to ASC indicated a higher pepsin extraction rate than ASC, which was confirmed by the higher yield of PSC (16.17 ± 0.21%) than of ASC (10.45 ± 0.33%). Enzymatic proteolysis was performed by digesting the cross-links in the peptide region at the end of the collagen molecule, and hence minor disintegrated smaller peptide fragments (Figure 1A, lane 2) were present in the PSC; however, these did not alter the integrity of the triple helix [27].

The crude peptide obtained after enzymatic digestion was purified by Sephadex G-50 to obtain four fractions of squid crude peptide (SCP1–SCP4) (Figure 1B), and the electrophoretic profiles of SCPs (SCP1–SCP4) (Figure 1C) demonstrated the lack of bands exceeding 20 kDa, indicating complete enzymatic hydrolysis of collagen into smaller molecules. For SCP1 and SCP4, distinct bands at 15 kDa and lighter bands at 12 kDa were observed, respectively. Based on the molecular weight, low content, high peak value, and color analysis in the NCBI database, SCP1 and SCP4 were potentially identified as cytochrome C proteins. Notably, SCP4, a component of SCP1, stayed attached to the dextran gel and was later released during elution. No bands were detected for SCP2 and SCP3, indicating efficient collagen digestion under alkaline protease conditions with released peptide molecular weights below 10 kDa. It is postulated that the absorption rate of a peptide depends on its molecular weight [28,29], and thus, low MW peptides can be readily bioavailable to the human body. Moreover, the UV absorption intensity of SCP3 was significantly greater than that of SCP2 in the plots. Consequently, the purified peptide SCP3 (hereafter referred to as SCP) was chosen for subsequent experiments. The MALDI-TOF mass spectrogram (Figure 1D) revealed a molecular mass range of 900–5000 Da for SCP, with a peak intensity of 1282 Da. The presence of a 1492 Da collagen peptide was observed as a secondary finding, allowing for an estimation of the molecular weight of SCP to be approximately 1.3 kDa.

### 2.2. Microstructure Analysis

**Figure 2:**
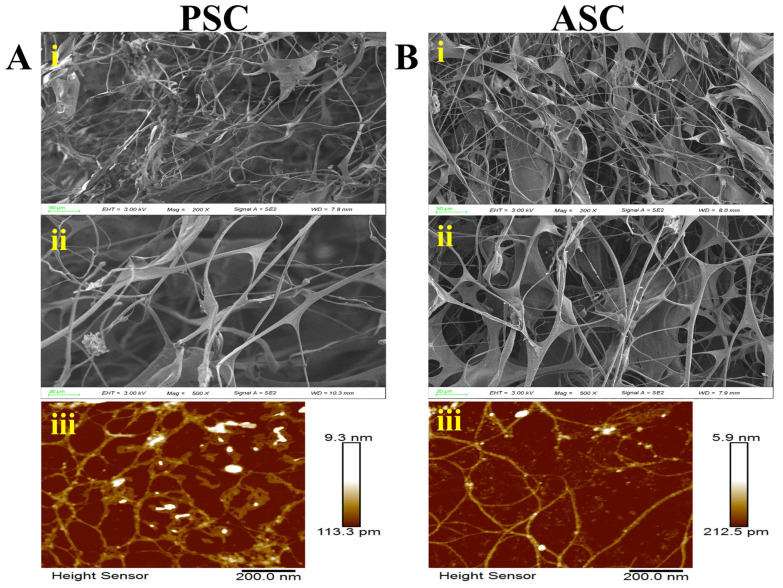
Scanning electron microscopic (SEM) structure and atomic force microscopic (AFM) structure of PSC (**A**) and ASC (**B**) isolated from squid skin. Letters (i,ii) represent SEM images, and (iii) represents AFM images. SEM image with different magnifications: (i) 50 μm, (ii) 20 μm. AFM image with magnifications of 200 nm (iii).

Figure 2 demonstrates the intricate fibrous layout of PSC and ASC obtained from squid skin, illustrating a complex multilayered composition of fibrous, lamellar, and sparse honeycomb porous collagen under scanning electron microscopy (Figure 2A,B(i, ii)). The interlinking of filaments with varying thicknesses demonstrated the fibrous nature of collagen. The fibers of ASC exhibited a finer, denser, and more porous structure compared to those of PSC. Additionally, the AFM results showed that the fiber bundle thickness of PSC (9.3 nm) was greater than that of ASC (5.9 nm) (Figure 2A,B(iii)), potentially due to an increased presence of terminal peptides resulting from pepsin treatment [30]. However, there were no significant alterations observed in the microstructure of collagens obtained through both methods. 

### 2.3. Thermogravimetric Analysis

**Figure 3:**
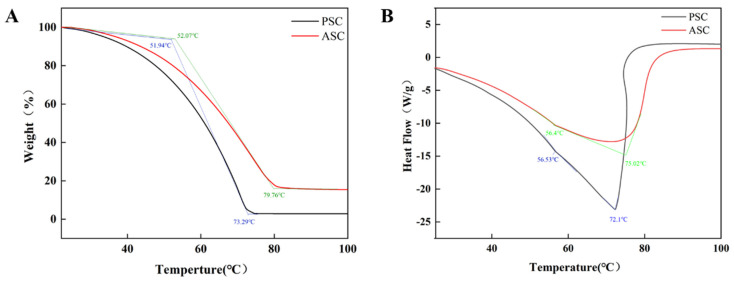
TG (**A**) and DSC (**B**) thermal denaturation curves of PSC and ASC from squid skin.

The thermal stability profile (Figure 3) revealed the denaturation temperature and melting temperature of the collagen. The thermogravimetric analysis (Figure 3A) demonstrated that the initial decomposition temperature of collagen was measured to be 20 °C. The contraction temperature of collagen is typically correlated with the onset temperature of weight loss. Collagen contraction occurs as a result of the disruption of cross-linking bonds between polypeptide chains, such as pyridinoline [31]. The heat absorption peak observed at 52 °C in the DSC thermogram (Figure 3B) was attributed to the denaturation of collagen, revealing a notably higher denaturation temperature in comparison to that of pig skin collagen (37 °C) [32]. The melting temperature of collagen ranged from 72.1 °C to 75.02 °C and was associated with the occurrence of peptide chain cleavage. The correlation between the thermal stability of collagen and the ambient temperature of the species’ habitat has been discovered to be significant [33]. This intriguing finding also extends to squid, a species renowned for its remarkable ability to withstand high temperatures. The denaturation temperature of collagen is reported to be determined by the degree of hydroxylation and specific amino acid residues (e.g., Gly-Pro-Hyp). Additionally, substituent amino acids play a significant role in the thermal properties of collagen [34]. In the present study, the squid skin collagen exhibited higher thermal stability and lower thermolysis due to its elevated iminic acid content as well as pyridine content [31].

### 2.4. Characteristic Absorption Analysis

**Figure 4:**
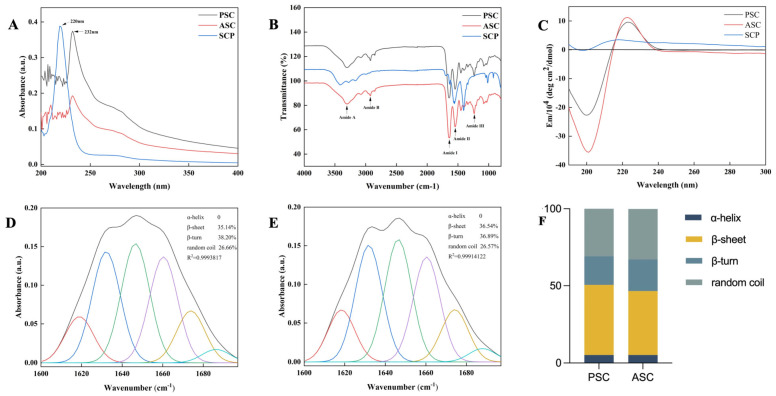
UV-vis spectra (**A**), FTIR spectra (**B**), and CD spectra (**C**) of PSC, ASC, and SCP from squid skin. The secondary structure of PSC (**D**) and ASC (**E**) was determined by Gaussian deconvolution analysis of the amide I region. (**F**) Percentage of PSC and ASC secondary structure resolved by CDNN software version 2.1.

The UV absorption spectra of PSC, ASC, and SCP at 200–400 nm (Figure 4A) exhibited a prominent absorption peak at 220 nm for SCP and at 232 nm for ASC and PSC, indicating a substantial glycine content (with maximum absorbance observed at 216.8 nm) as well as the presence of peptide chains in the collagen matrix (at around 230 nm). The collagen peptide SCP exhibited maximum absorbance at 220 nm, which differs from that of collagen, potentially attributed to the exposure of functional groups such as C=O, -COOH, and CONH_2_ in the polypeptide chain of collagen after proteolysis [35]. However, no absorption peak at 280 nm was observed in collagen, which confirmed the absence of non-collagen protein. The results of this study demonstrated that the extracted collagen exhibited a low abundance of tyrosine and tryptophan residues, as well as sulfur-containing amino acids like cysteine [36,37], which was further corroborated by the corresponding amino acid content presented in Table 1. Consequently, it can be inferred that the extracted collagen possessed a high degree of purity [27,38]. Furthermore, the purity levels of PSC and ASC were determined to be 92.77 ± 0.75% and 92.44 ± 5.61%, respectively. The maximum absorption of pure collagen (99%) was reported to be 230 nm [39]. The significantly higher absorption intensity observed in PSC compared to ASC may be attributed to the enzymatically cleaved peptides present in PSC.

The secondary structures of collagen (PSC and ASC) and peptide (SCP) were further evaluated using infrared spectroscopy, as depicted in Figure 4B. The characteristic peaks corresponding to amides A, B, I, II, and III exhibited similarities with PSC and ASC, consistent with previously reported collagens derived from diverse marine sources [40,41,42,43]. The positions of the amide bands and the arrangement of the dependent groups are presented in Table 2. The amide A band typically exhibits a free N-H stretching vibration [44] within the range of 3400 to 3440 cm^−1^. The amide A band of SCP (3411 cm^−1^) was attributed to the hydrogen bonding of the N-H moiety in peptide synthesis [4]. However, the amide A band of PSC and ASC was observed at approximately 3300 cm^−1^, indicating a downshift in frequency for the NH groups involved in hydrogen bonding [44]. The presence of this absorption peak also signifies the preservation of the characteristic methylene group in the tertiary structure of collagen, thereby indicating the integrity of its tertiary structure. The wavelength of amide B at 2924 cm^−1^ indicated CH_2_ group asymmetric stretching [45]. The amide B band of SCP was observed at a wavenumber of 3161 cm^−1^. The absorption of the amide I peak was attributed to the stretching of C=O bonds, along with the bending vibrations exhibited by NH groups [46]. The amide I peak was observed at 1640 cm^−1^, 1643 cm^−1^, and 1633 cm^−1^ for PSC, ASC, and SCP, respectively; the amide I band of SCP was positioned at a lower level, suggesting that the C=O stretching vibration of the peptide bond may alter the triple-helix structure. The wavenumbers at the amide II bands of PSC and ASC (1539 cm^−1^) were observed to be lower, indicating a higher degree of hydrogen bonding [47] in collagen derived from squid compared to the typical absorption range at the amide II band position (1550–1600 cm^−1^). The amide II band of SCP was observed at 1562 cm^−1^, displaying a characteristic absorption peak. The presence of hydrogen bonding was indicated by the observation of the amide III band at 1236 cm^−1^. The IR ratio of the band positions at 1236 cm^−1^ (amide III) and 1539 cm^−1^ (amide II) was determined to be 1.24, indicating that both PSC and ASC maintained the intact triple-helix structure [48]. The amide III band of SCP was detected at 1408 cm^−1^, likely indicating the participation of the N-H group in peptide synthesis.

Circular dichroism diagrams were employed to verify the secondary structure. According to the study, collagen typically exhibited a characteristic positive peak at approximately 220 nm and a characteristic negative peak at around 198 nm on circular dichroism spectra. The degeneration of collagen indicated the disappearance of the characteristic positive peak [49], concluding with a complete disruption of both collagen and triple-helix structures. As depicted in Figure 4C, PSC and ASC displayed significant characteristic positive peaks at 223 nm, along with strong negative peaks at 200 nm and 201 nm, respectively. This observation is consistent with the absorption pattern exhibited by collagen extracted from tilapia and Pacific cod [50]. The negative absorption peak (0.42 for PSC and 0.32 for ASC) indicated intact triple-helix structures of both collagens. [51]. In contrast, the lack of a positive absorption peak at 220 nm and a negative peak at 198 nm of SCP indicated structural integrity loss of the triple-helix structure [49].

**Table 2:** The amide band positions in FTIR spectra of collagen PSC, ASC, and collagen peptide SCP from squid skin.

Band	Frequencies of Amide Bands [cm^−1^]			Assignment
	PSC	ASC	SCP	
Amide A	3307	3303	3411	NH stretching [44]
Amide B	2924	2924	3161	CH_2_ asymmetrical stretching [52]
Amide I	1640	1643	1633	C=O stretch Hydrogen bond coupled with COO- [46]
Amide II	1539	1539	1562	N-H bending coupled with CN stretching [53]
Amide III	1236	1236	1408	

The amide I band primarily reflects the secondary structure of collagen, and the proportions of PSC (Figure 4D) and ASC (Figure 4E) secondary structures were determined through Gaussian fitting analysis. The β-sheet content of PSC and ASC was found to be 35.14% and 36.54%, respectively, while the β-turn content was measured at 38.20% for PSC and 36.89% for ASC; additionally, both samples displayed random coil formation of approximately 26.66% for PSC and 26.57% for ASC. However, the absence of an α-helix structure was not observed in either type of collagen, potentially attributed to the higher glycine content inherent in collagen [54]. The secondary structure proportion (Figure 4F) was determined using molecular circular dichroism analysis with CDNN software version 2.1 (Applied Photophysics Ltd., Surrey, UK), revealing the lowest proportion of α-helices. These findings suggest that both PSC and ASC extraction methods did not impact the collagen’s secondary structure, indicating the suitability of the pepsin dissolution method for collagen extraction.

### 2.5. Amino Acid Composition Pattern

The amino acid composition of collagen and collagen peptide, expressed as the number of amino acid residues per 1000 amino acid residues, is presented in Table 1. The unique repetitive amino acid structure (Gly-Pro-Hyp) found in collagen resulted in a high glycine content, making up 32.87% and 33.51% of the total amino acid composition in PSC and ASC, respectively [55]. Next to glycine, the major amino acids found in collagen were hydroxyproline, proline, alanine, and glutamic acid. This result was consistent with the amino acid composition of collagens from other fish skin sources, such as shark skin and cartilage, tilapia scales and skin, and starfish [56,57,58]. The proline and hydroxyproline residues within collagen collectively contributed imino acid contents of 21.34% and 20.77% for PSC and ASC exhibiting iminic acid, respectively. The presence of the pyrrolidine ring in imino acid contributes to the reinforcement of collagen’s triple-helical structure, making imino acid content a reliable indicator for evaluating collagen’s thermal stability [59]. The amino acid composition patterns between PSC and ASC showed a general resemblance; however, PSC demonstrated a comparatively elevated abundance of most amino acids. This dissimilarity can be ascribed to the utilization of an enzymatic solubilization technique that efficiently cleaved the intermolecular cross-links within the terminal peptide region of collagen molecules, leading to the production of multiple smaller peptides [60]. The amino acid composition pattern of collagen peptide SCP closely resembled that of its parent collagen PSC, exhibiting the highest proportion of glycine residues followed by proline. In contrast to PSC, methionine was not detected in SCP, likely due to its low content and removal during enzyme digestion as a result of the relatively low ratio of methionine in PSC (0.17%). The hydrophobic amino acids, namely alanine, threonine, valine, proline, isoleucine, leucine, methionine, and phenylalanine, collectively accounted for 28.10%, 28.28%, and 32.61% of the overall amino acid composition in PSC, ASC, and SCP, respectively. Peptides exhibiting ACE-inhibitory activity were commonly characterized by the presence of hydrophobic amino acids at the C-terminal region [61]. The hydrophobic nature of these amino acids facilitates their interaction with the active site of ACE and offers antimicrobial activity [62]. Therefore, squid-derived collagen with a high concentration of hydrophobic amino acids represented an exceptional source for biologically active peptides.

### 2.6. De Novo Sequencing

A total of 238 peptides were identified from the squid database, out of which 43 collagen peptides exhibited scores exceeding 300 points, while 139 peptides scored between 200 and 300 points. In principle, a score above 200 points indicated higher credibility. Among the subset of peptides with scores surpassing 300, it was found that thirty-two shared similarities with previously identified type I collagen. Table 3 presents partial amino acid sequence diagrams for SCP. The consistent occurrence of glycine and proline in the polypeptide sequence, along with the repetitive presence of proline at positions “X” and “Y”, further confirmed the inherent triple-helix structure found in collagen polypeptides containing (Gly-X-Y) repeats. The above results were also supported by the amino acid composition.

### 2.7. Regulation of Proliferation and Migration of HFF Cells

#### 2.7.1. Cell Proliferation

**Figure 5:**
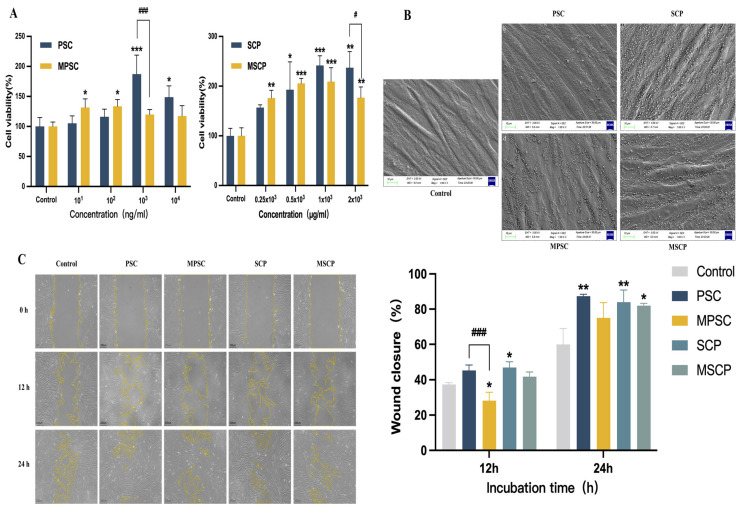
Cell proliferation rate of HFF-1 cells cultured with different concentrations of collagen and collagen peptide (**A**) for 24 h. Scanning electron microscopy (**B**) of HFF-1 cells treated by collagen and collagen peptide for 24 h. Effect of PMSM on migration ability of HFF-1 (scale bar: 10 μm). (**C**) Cell mobility and migration maps of wounds treated with collagen and collagen peptides in scratch tests, respectively. Images were taken at 0, 12, and 24 h. Scale: 200 μm. Data represent mean ± SD, n = 3. *, **, and *** represent *p* < 0.05, *p* < 0.01, and *p* < 0.001 compared with the control group. # and ### indicate *p* < 0.05 and *p* < 0.001; the difference between the two groups is statistically significant. Note: PSC: squid-derived pepsin-soluble collagen; MPSC: market-derived bovine collagen; SCP: squid-derived collagen peptides; MSCP: market-derived ichthyosis collagen tripeptide, the same below.

The cell proliferation rate affected by collagen concentration is shown in Figure 5A. Within a specific concentration range, the highest cell viability was observed at 10^3^ ng/mL for PSC and at 10^2^ ng/mL for market bovine Achilles tendon collagen (MPSC). Notably, PSC exhibited significantly higher proliferation than MPSC at 10^3^ and 10^4^ ng/mL concentrations. A higher proliferation rate of HFF-1 cells was observed at 1 × 10^3^ μg/mL collagen peptide for market fish scale collagen tripeptide (MSCP). The results show that collagen peptide demonstrates markedly higher proliferative effects than collagen protein at various concentrations. This could be due to the extensive direct absorption of collagen peptides [63] by cells without requiring enzymatic hydrolysis. The proliferative results concluded that both collagen and collagen peptides promoted the proliferation of HFF-1 cells.

After 24 h of drug treatment, cells were fixed and subsequently subjected to gold spraying after drying for scanning electron microscopy (SEM). The SEM image (Figure 5B) revealed slender fibers with a closely arranged morphology, abundant cytoplasm, and intact cell membrane of HFF-1 cells. Interestingly, the addition of the drug did not alter the original cell morphology.

#### 2.7.2. Cell migration

The monolayer of HFF-1 cells was artificially wounded to create an artificial wound model. Following 12 h of drug treatment, a significant reduction in the width of the scratch was observed, accompanied by clear migration trajectories of HFF-1 cells toward the wound area. This intriguing movement pattern suggested that collagen and collagen peptides possessed remarkable migration- and proliferation-promoting capabilities, thereby promoting tissue repair. Figure 5C demonstrates that collagen and collagen peptides increased the migration distance of HFF-1 cells by 87.37%, 75.08%, 83.97%, and 82% respectively, which was significantly higher than that observed in the control group (59.93%, *p* < 0.01).

Notably, the presence of PSC at a concentration of 10^3^ ng/mL resulted in the highest cell healing rate. The migration to the injury site was significantly enhanced in the presence of collagen and collagen peptides compared to the absence of any other drug action in the medium. MPSC, an animal-derived collagen with a mobility of 75.08%, exhibited higher efficiency than the blank control group (15.14%), indicating that marine-derived collagen outperformed animal-derived collagen. In conclusion, both collagen and collagen peptides effectively promoted cell migration into the artificial wound. The migration of fibroblasts into damaged tissues plays a crucial role in the process of tissue remodeling [64]. Cell scratch assays revealed that collagen and collagen peptide could induce the migration of HFF-1 cells, possibly through the chemotactic effect of collagen, which facilitated the movement of HFF-1 cells toward the site of artificial wound [65]. Moreover, the presence of abundant amino acid residues, cytokines, and other essential nutrients in collagen promoted cell growth and proliferation [66].

### 2.8. Relative Protein Expression of HFF Cells 

**Figure 6:**
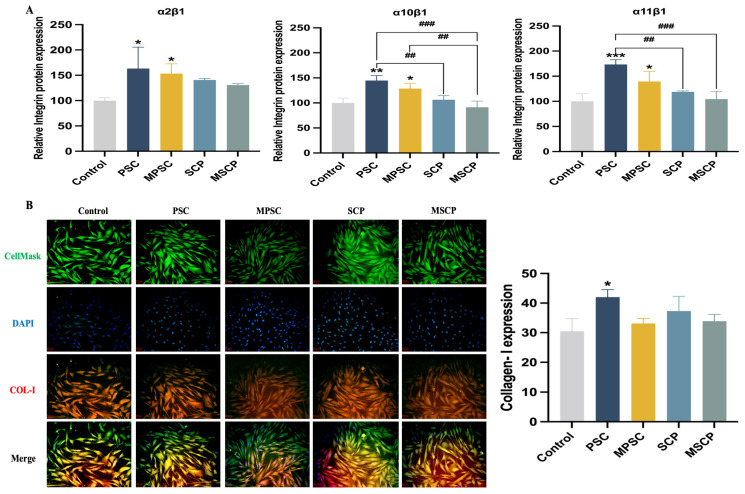
Relative protein expression of HFF-1 cells cultured with collagen and collagen peptide for 24 h. (**A**) Quantification of integrin receptors α2β1, α10β1, and α11β1 by ELISA. (**B**) Fluorescence microscopy images, scale: 50 μm, and quantification of COL-I by ELISA. Data are expressed as mean ± SD, n = 3. *, **, and *** mean *p* < 0.05, *p* < 0.01, and *p* < 0.001 compared with the control group. ## and ### indicate that *p* < 0.001 and *p* < 0.001 are statistically significant between the two groups.

Integrin α_1_, α_2_, α_10_, and α_11_ bind to the integrin β_1_ subunit to form collagen receptors [67]. Recent findings have emphasized the role of collagen-binding integrins in wound fibroblast function [68]. Integrin α_2_β_1_ is expressed by various cell types, including platelets and fibroblasts. The distribution of integrin α_10_β_1_ is limited to mesenchymal stem cells and chondrocytes, while integrin α_11_β_1_ is restricted to mesenchymal stem cells and a subset of fibroblasts [69]. Integrin α_11_β_1_ plays a crucial role in collagen reorganization by promoting collagen remodeling and upregulating its expression. This distribution was also confirmed by the results of integrin expression (Figure 6A). Compared with the control group, drug treatment led to a relative increase in the number of integrin receptor subunits in HFF-1 cells, with statistically significant changes observed in the expression of integrin receptors in both collagen PSC and MPSC (*p* < 0.05). The protein expression of integrin α_2_β_1_ and α_11_β_1_ was comparable but higher than that of α_10_β_1_. The expression level of integrin α_10_β_1_ in HFF-1 cells was significantly lower and did not exhibit increased affinity for the α_10_β_1_ integrin receptor in the presence of collagen peptide. However, the expression level of the receptor in HFF-1 cells treated with collagen was higher than that with collagen peptide. Although there was an upregulation of integrin receptor expression in the presence of collagen peptides compared to controls, it did not reach statistical significance. Marine-derived collagen PSC demonstrated elevated expression levels of α_2_β_1_, α_10_β_1_, and α_11_β_1_ integrin receptors when compared to bovine collagen. Rybinski et al. [70] established the crucial role played by collagen-binding integrins in fibroblasts, particularly during wound healing and fibrosis processes. Collagen and its peptides can enhance binding between integrin receptors and promote their surface expression on HFF-1 cells, thereby contributing to fibrosis. The network of collagen fibers imparts elasticity to the skin, with type I collagen playing a pivotal role. The analysis of type I collagen levels in skin fibroblasts (red fluorescence) and ELISA results (Figure 6B) revealed that the presence of collagen (10^3^ ng/mL) and collagen peptide (1 × 10^3^ µg/mL) significantly upregulated the expression of type I collagen (COL-I), with statistical significance observed for collagen compared to the control group. This finding further substantiated that both collagen and collagen peptides could enhance the synthesis of collagen by skin fibroblasts, thereby exerting anti-aging effects. Importantly, this outcome aligned with integrin receptor expression findings and underscored how specific binding interactions between cellular integrin receptors and collagens promoted the subsequent upregulation of collagens.

### 2.9. Protective Effects of Collagen Peptides against H_2_O_2_-Induced Fibroblast Injury

**Figure 7:**
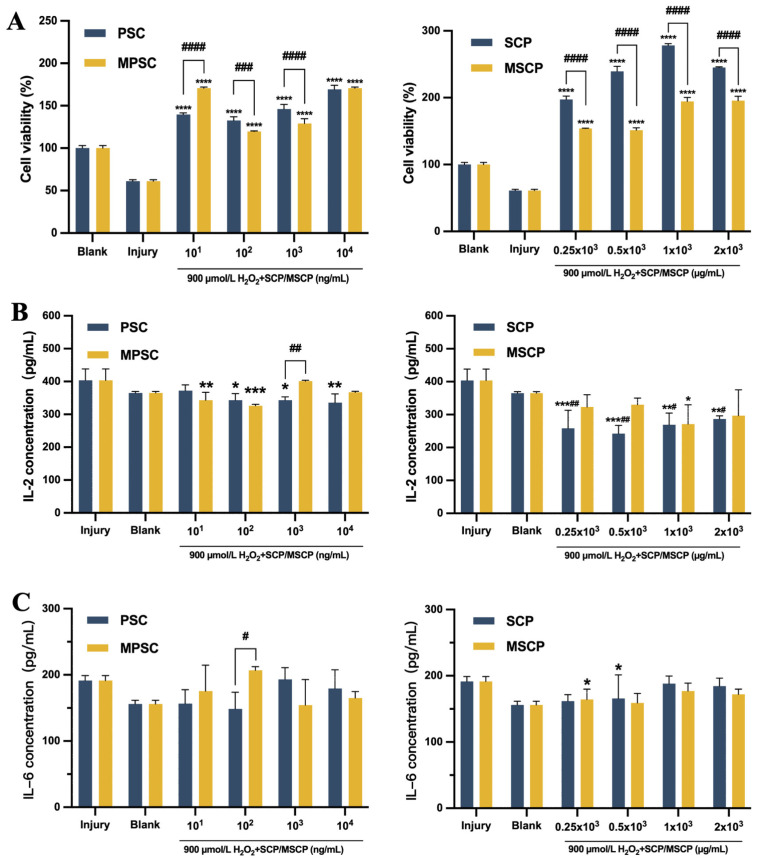
Effects of different concentrations of collagen and collagen peptide on the cell viability (**A**) of HFF-1 cells damaged by H_2_O_2_ for 24 h. Expression of inflammatory factors IL-2 (**B**) and IL-6 (**C**) in HFF-1 cells damaged by H_2_O_2_ cultured with different concentrations of collagen and collagen peptide. *, **, ***, and **** indicate *p* < 0.05, *p* < 0.01, *p* < 0.001, and *p* < 0.0001, respectively, compared with the injury control group. # and ## mean *p* < 0.05 and *p* < 0.01 compared with the blank control group, respectively. #, ##, ###, and #### indicate that *p* < 0.05, *p* < 0.01, *p* < 0.001, and *p* < 0.0001 are statistically significant between the two groups. Values are mean ± SD, n = 3.3.

In human dermal fibroblasts, treatment with H_2_O_2_ significantly augmented skin damage. Following H_2_O_2_-induced injury and subsequent 24 h culture in the presence of collagen and collagen peptide (Figure 7A), the cell proliferation rate was markedly higher compared to that in the injured group. Cell viability was enhanced under the protection of 10^2^ ng/mL and 10^3^ ng/mL collagen, surpassing that of the blank control group, with PSC demonstrating superior protective effects over MPSC. Remarkably, high cell viability was observed in the presence of 10^4^ ng/mL collagen. Injured cells demonstrated a noteworthy enhancement in cell viability upon exposure to SCP, surpassing the cell viability observed with MPSC. Under a concentration gradient, injured cells cultured in both 1 × 10^3^ µg/mL SCP and MSCP displayed a high proliferation rate consistent with normal HFF-1 cells’ proliferation rate. Collagen protein and collagen peptide demonstrated alleviating effects on H_2_O_2_-induced injury in HFF-1 cells. The protective impact of these substances on severe HFF-1 damage induced by H_2_O_2_ led to increased cellular activity and further promoted wound healing. Studies have revealed that fibroblasts can produce interleukin-6 (IL-6) upon stimulation and participate in inflammatory responses, while interleukin-6 (IL-2) can activate immune cells’ function [71], thereby accelerating wound healing. The levels of the inflammatory cytokine IL-2 are shown in Figure 7B, revealing a significant reduction in its expression compared to that in the injured group. Notably, at a concentration of 10^4^ ng/mL for PSC and 0.5 × 10^3^ µg/mL for SCP, the expression of IL-2 was measured as 335 pg/mL and 242 pg/mL respectively, both significantly lower than that observed in the injured group (400 pg/mL). As expected, collagen and collagen peptide treatment resulted in low secretion of IL-6 after stimulation with collagen (Figure 7C). Importantly, both collagen and collagen peptide exhibited superior protective effects on injured HFF-1 cells compared to those on the blank group and injury control group. This further supported their antioxidant properties as well as their ability to reduce reactive oxygen load when applied to wound sites, thereby accelerating tissue healing [72]. In conclusion, collagen and collagen peptides effectively inhibit inflammatory factor secretion while utilizing a certain level of cellular protection that promotes cell proliferation.

## 3. Materials and Methods

### 3.1. Chemicals

Sodium hydroxide (NaOH), hydrochloric acid (HCl), glacial acetic acid, sodium chloride (NaCl), ammonium bicarbonate, sodium citrate, and 3, 5-dimethyl-4-hydroxycinnamic acid (SA) were obtained from Sinopharm Chemical Reagent Co., Ltd. (Shanghai, China). Porcine gastric mucosa pepsin (3000–3500 NFU/g) and alkaline protease (≥20,000 KFU/g) were purchased from Solarbio Biotechnology Co., Ltd. (Beijing, China). Phosphate buffer solution (PBS) was obtained from Servicebio Technology Co., Ltd. (Wuhan, China). N-butanol, absolute ethanol, methanol, and acetonitrile were procured from Titan Technology Ltd. (Shanghai, China). Coomassie brilliant blue R-250 was purchased from Sigma-Aldrich Corporation (St. Louis, MO, USA). An Omni-EasyTM One-Step PAGE Gel Fast Preparation Kit (7.5%, 12.5%) and dual-color protein standard marker with a molecular weight of 10–250 kDa were purchased from Yamei Biopharmaceutical Technology Co., Ltd. (Shanghai, China). Sephadex G-50 was acquired from Yuanye Biotechnology Ltd. (Shanghai, China). Unless specified otherwise, all chemical reagents utilized in the experimental group were of high analytical purity.

### 3.2. Preparation of Squid Collagen and Collagen Peptides

#### 3.2.1. Preparation of Pepsin-Soluble and Acid-Soluble Collagen

The raw materials, squid (*Symplectoteuthis oualaniensis*), were freshly collected from the northwestern Indian Ocean region and stored at −20 °C in the School of Fisheries, Shanghai Ocean University, China. After being thawed at room temperature, the squid skin tissue was preserved after removing the head, tail, viscera, and other parts of the squid. The skin tissues were thoroughly rinsed with water, subsequently drained, and placed in polyethylene bags for storage at −20 °C. The entire process was conducted under controlled conditions at 4 °C.

**Figure 8:**
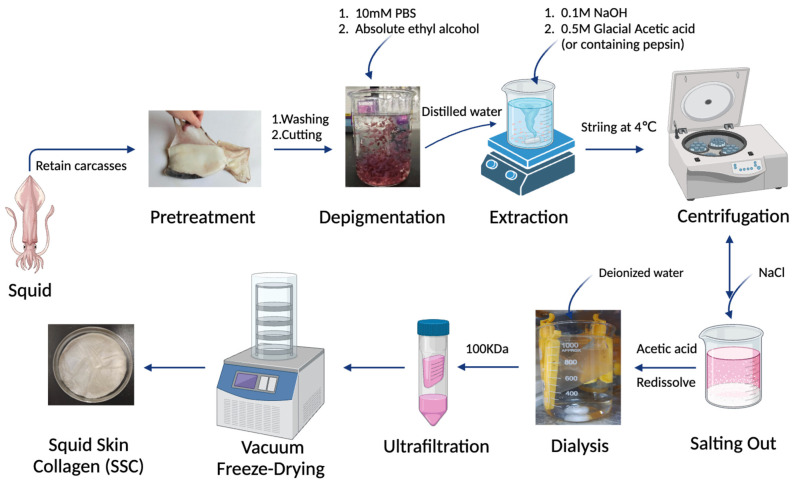
Schematic diagram of squid skin collagen extraction steps.

The squid skin was thawed at 4 °C, cut into pieces measuring 0.5 × 0.5 cm, and subsequently immersed in 0.01 mol/L PBS (pH 7.2) at a solution ratio of 1:10 (*w*/*v*) for 12 h. The skin samples were depigmented by immersing them in anhydrous ethanol for 12 h. Subsequently, the ethanol-treated samples were subjected to immersion in a 0.1 mol/L NaOH solution at a ratio of 1:10 (*w*/*v*) for 48 h with continuous agitation, aiming to eliminate heteroproteins from the non-collagenous constituents. The NaOH solution was replaced every 6–8 h, followed by a subsequent 24 h treatment with 10% n-butanol for further degreasing purposes. The solution was replaced every 6 h and rinsed thoroughly with deionized water until reaching a neutral pH. The pretreated skin tissue was homogenized and treated with 0.5 mol/L glacial acetic acid containing 1% (*w*/*w*) pepsin at a solute ratio of 1:20 (*w*/*v*). The collagen from the skin was solubilized in glacial acetic acid and extracted at 4 °C for 48 h under constant stirring. The supernatant was collected by centrifugation at 10,000× *g* for 30 min. Subsequently, the supernatant was subjected to salting out by adding NaCl until saturation. The precipitate was collected by centrifugation at 10,000× *g* for 30 min and subsequently re-dissolved in a 0.5 mol/L glacial acetic acid solution. It was then dialyzed against deionized water for two days using dialysis bags with a molecular weight cut-off (MWCO) of 100 kDa (Shanghai Yuanye Bio-Technology Co., Ltd., Shanghai, China). The purified collagen was named as pepsin-soluble collagen (PSC). The PSC was transferred to a 50 mL ultrafiltration tube with MWCO of 100 kDa and centrifuged at 2000× *g* for 30 min, and the purified collagen was identified as U-PSC. The extraction process of acid-soluble collagen (ASC) was slightly different from that of PSC, where the homogenate was added to 0.5 mol/L acetic acid solution not containing 1% (*w*/*w*) pepsin and followed the same procedure as mentioned for PSC. The entire process was conducted at a temperature of 4 °C. Following dialysis, both PSC and ASC were subjected to freeze-drying at −70 °C and 2 Pa for 48 h using a vacuum freeze dryer (Labconco Corporation, Kansas City, MO, USA). The detailed process for extracting collagen from squid skin is illustrated in Figure 8.

#### 3.2.2. Preparation and Purification of Collagen Peptides

**Figure 9:**
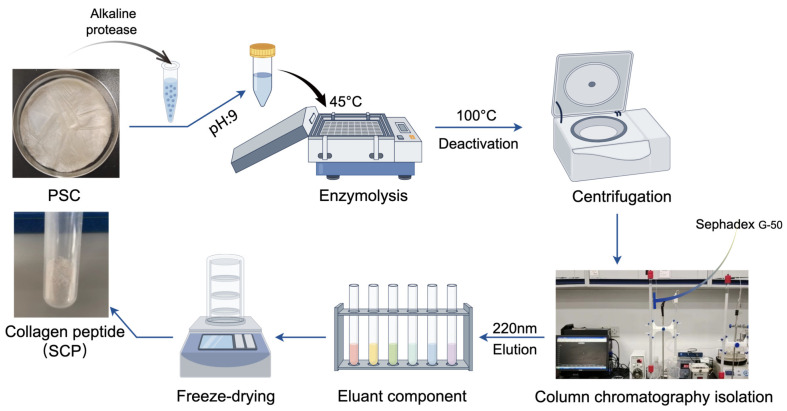
Schematic diagram of squid skin collagen peptide extraction steps.

The PSC collagen (1 g) was dissolved in a 10 mL 0.5 mol/L acetic acid solution and the pH was adjusted to 9. The solution was preheated at 45 °C for 15 min in a constant-temperature oscillating incubator, followed by enzymatic digestion using 0.2% (*w*/*w*) alkaline protease at 45 °C for 45 min. The enzyme digest was promptly subjected to a 100 °C water bath for 10 min to achieve enzyme inactivation. After being cooled to room temperature, the squid crude peptide (SCP) was collected through centrifugation at 10,000× *g* for 30 min. Subsequently, the SCP (0.5 mL) underwent purification by gel filtration chromatography. Briefly, Sephadex G-50 was soaked in distilled water for 48 h to allow it to fully dissolve, slowly injected into the column, and left to stand overnight. The solution was then equilibrated with ultrapure water to A230 less than 0.05 for baseline correction and eluted at a flow rate of 1 mL/min. The spectroscopic chromatogram was identified by UV absorption spectroscopy at 220 nm. The major sections were collected and subjected to lyophilization for subsequent analysis. The process flow for the preparation of collagen peptides from squid collagen is depicted in Figure 9.

### 3.3. Molecular Pattern Analysis

The molecular patterns of collagen and peptide were characterized using sodium dodecyl sulfate-polyacrylamide gel electrophoresis (SDS-PAGE). PSC, ASC, and SCP were dissolved in a 4:1 ratio in a 5 × SDS-PAGE loading buffer and mixed thoroughly using a vortex mixer (Beijing Tiangen Biotechnology Co., Ltd., Beijing, China). Following dialysis and ultrafiltration, PSC and ASC underwent immediate electrophoresis. The quality of pretreatment samples and the final extracted collagen volume remained consistent throughout the extraction process. Similarly, SCP was immediately subjected to electrophoresis upon collection. The samples were subjected to heating at 100 °C for 5 min in a metal bath, followed by transfer of the heated samples to a handheld centrifuge for centrifugation lasting 1 min. A polyacrylamide gel was prepared with a thickness of 1 mm, consisting of a stacked gel containing 4.5% and a resolved gel containing 7.5% and 12.5%, following the instructions provided in the Omni-EasyTM One-Step PAGE Gel Fast Preparation Kit description. Then, 5 µL of the protein standard (10–250 kDa) and 10 µL of the protein sample were loaded into the gel well and subjected to electrophoresis in 1× Tris/glycine/SDS buffer for 45 min at a constant voltage of 200 V. Following electrophoresis, the gel was stained with staining reagent comprising 0.25% (*w*/*v*) Coomassie brilliant blue R250, 45% (*v*/*v*) methanol, 10% (*v*/*v*) acetic acid, and 45% (*v*/*v*) distilled water to visualize the distinct protein bands. The excess stains were removed using a destaining reagent composed of ethanol, acetic acid, and distilled water in a ratio of 2:1:7. The images were captured using the gel imager system (Gensens 2100(T), Shanghai Clinx Science Instruments Co., Ltd., Shanghai, China). The molecular weights of the collagen peptides SCP were predicted based on matrix-assisted laser-resolved time mass spectrometry (MALDI-TOF). The SCP was diluted with distilled water, and the samples were pretreated with 50% (*v*/*v*) acetonitrile at a volume ratio of 2:1. A concentration of 15 mg/mL 3,5-dimethoxy-4-hydroxycinnamic acid (SA) solution was used as the MALDI substrate, a 1 µL sample spot was placed on a MTP384 stainless steel non-polished target plate and air dried naturally, and then 1 µL of SA substrate solution was added to cover the sample spot and air dried again. Subsequently, the laser was focused on the sample using an 8.02 Kv lens, and a MALDI mass spectrometer (Brukern Ultraflextreme, Beijing, China) with an accelerating voltage of 20 Kv was used to perform MALDI-TOF-MS analysis in 10 different sample deposition regions. The obtained MALDI spectra were analyzed by FlexAnalysis 3.4 software for processing.

### 3.4. Scanning Electron Microscopy (SEM) and Atomic Force Microscopy (AFM)

The collagen’s microscopic morphology was observed using the Zeiss Gemini SEM 300, an ultra-high-resolution field emission scanning electron microscope. The collagen samples were directly affixed onto conductive adhesive tape and subsequently coated with a 10 mA gold layer for 45 s using a Quorum SC7620 sputter coater (Quorum Technologies, Lewes, UK). The collagen samples were captured using a ZEISS Gemini SEM 300 scanning electron microscope (Carl ZEISS Ltd., Oberkochen, Germany) operating at accelerating voltages of 3 kV and 15 kV for spectral mapping shots. The utilized magnifications were 200 µm, 100 µm, and 50 µm, respectively. An SE2 secondary electron detector was employed for topography detection. The fiber arrangement and surface properties of PSC and ASC were observed using an atomic force microscope (Bruker Dimension Icon, KL, Germany). The freshly exposed white mica flakes were coated with 20 µL of collagen solution and air dried at room temperature for 2 h. Subsequently, the mica with attached collagen was carefully positioned on the sample release table. The imaging was conducted using NanoScope Analysis 1.8 software (Bruker Corporation, Kaiserslautern, Germany) at a resolution of 512 × 512 pixels and a scanning rate of 1 Hz.

### 3.5. Thermogravimetric Differential Scanning Calorimetry (TG-DSC)

The thermal stability of collagen was assessed using a simultaneous thermal analyzer (TAQ600, Hercules, CA, USA). PSC and ASC were dissolved in acetic acid and dialyzed in deionized water until the collagen samples reached a neutral pH. The collagen solution (1 mL) was uniformly placed in an alumina crucible and subjected to heating from 20 °C to 100 °C under a nitrogen atmosphere (purity ≥ 99.9%) at a specified heating rate of 20 °C/min for the absorption and exothermic curves as well as thermogravimetric measurements. The reference was an aluminum disk that remained empty. The two collagens were repeated three times under identical conditions.

### 3.6. UV–Visible Spectroscopy (UV-Vis)

The maximum absorption peaks of collagen and peptide were determined to assess the purity of the proteins. The PSC, ASC, and SCP were prepared by dissolution in 0.5 M acetic acid to obtain a 0.1 mg/mL sample solution. The UV-vis absorption spectra were recorded in the range of 200 to 400 nm using a MAPADA spectrophotometer (Model UV-3000pc, Shanghai, China) at a scanning speed of 2 nm/s with intervals of 1 nm. A blank solution containing 0.5 M acetic acid was used for reference.

### 3.7. Fourier Transform Infrared Spectroscopy (FTIR)

The secondary structure of collagen was determined by acquiring FTIR spectra using a Fourier transform infrared spectrometer (L1050050 Spotlight 400, PerkinElmer Co., Hopkinton, MA, USA). An initial air background was employed for the acquisition of FTIR spectra. The instrument plate was loaded with 2 mg of collagen and peptide, and absorption spectra were recorded in the range of 600 to 4000 cm^−1^ in absorption mode at 4 cm^−1^ intervals for 32 scans using an attenuated total reflection (ATR) accessory. Interferences from H_2_O and CO_2_ were effectively eliminated during the process. The secondary structure of collagen was analyzed using PeakFit Version 4 software (SeaSolve software Inc., Framingham, MA, USA). The peak area of each secondary structure was obtained by Gaussian fitting to the amide I region (1600–1700 cm^−1^), while the percentage of secondary structure was calculated as the ratio between the peak area of each secondary structure and the total peak area encompassing all secondary structures. The experiment was conducted in triplicate with consistent conditions for each sample.

### 3.8. Circular Dichroism (CD) Spectra

A circular dichroism spectrometer (BRIGHTTIME Chirascan, Applied Photophysics Ltd., Charlotte, NC, USA) was employed to determine the spectra of collagen and collagen peptides. First, 0.5 mg of PSC, ASC, and SCP were accurately weighed in 1 mL of 0.5 mol/L acetic acid, followed by complete dissolution at 4 °C overnight to obtain a collagen and peptide sample solution with a concentration of 0.5 mg/mL. The quartz cuvette was thoroughly washed with an appropriate washing solution and subsequently rinsed with a glacial acetic acid solution (0.5 mol/L). Subsequently, the solutions containing PSC, ASC, and SCP were sequentially loaded into the quartz cuvette for CD scanning within the wavelength range of 190–300 nm at a scanning rate of 100 nm/min and maintained at a temperature of 25 °C, while using glacial acetic acid (0.5 mol/L) as the background.

### 3.9. Amino Acid Composition

The analysis of the amino acid content of collagen and collagen peptide was conducted using an ultra-high-speed automatic amino acid analyzer (LA8080, Hitachi, Tokyo, Japan). Referring to the national standard method, samples of PSC, ASC, and SCP were placed in the extraction hydrolysis tube, and 10 mL of 6 mol/L hydrochloric acid was added to the hydrolysis tube. The vacuum was removed, followed by a 30 s nitrogen charging process to facilitate blow sealing. The tubes were dried in an electric blast drying oven at 110 °C for 22 h. The hydrolysate was transferred to mini Petri dishes and dried under reduced pressure at 50 °C using a DZF368 6030 vacuum drying oven (Shanghai Shimo Medical Instrument Manufacturing Co., Ltd., Shanghai, China). The dried residue was eluted with deionized water until completely dissolved and dried again under reduced pressure until evaporation. The residue was fully dissolved in 1 mL of a pH 2.2 sodium citrate buffer solution with a concentration of 0.2 mol/L, followed by filtration through a 0.22 μm nylon membrane filter and subsequent transfer to a sample vial for analysis. Before analyzing the test samples, the amino acid analyzer was calibrated using standard reagents, and positive controls for all amino acids were employed as references. The identification of amino acid type and content in the samples was accomplished by comparing the retention time and peak area of the positive control peaks. The analysis of amino acid composition was repeated thrice.

### 3.10. Protein De Novo Sequencing

The collagen peptide SCP was dissolved thoroughly in 50 mM NH_4_HCO_3_ solution, and dithiothreitol (DTT) solution was added to reach a final concentration of 10 mmol/L. Then, the samples were heated in a water bath at 56 °C for 1 h. Subsequently, iodoacetamide solution (IAM) was added to reach a final concentration of 50 mmol/L, and the reaction was carried out for 40 min in a light-protected environment. The samples were desalted using a self-completion desalting column, and the solvent was dried up by centrifugation and concentrating in a vacuum at 45 °C. The processed samples were then analyzed by liquid–liquid mass spectrometry (LC-MS/MS) to obtain the raw mass spectrometry results as raw files. The peptide sequence analysis was conducted using the PEAKS Studio 10.6 de novo method, while the squid protein database was searched through Byonic.

### 3.11. Cell Proliferation Assay

HFF-1 cells were offered by Zhejiang Meisen science and technology Co., Ltd. (Jinhua, China) and cultured in a suspension containing DMEM with 15% fetal bovine serum (FBS), 1% p/s at 37 °C in an incubator with a humidified atmosphere of 5% CO_2_ (BB 150, Thermo Fisher Scientific, Waltham, MA, USA). HFF-1 cells were seeded in 96-well cell culture plates at a density of 5 × 10^4^ cells mL^−1^ when they reached 80% confluence. The cells were cultured for 24 h with collagen solution at final concentrations of 10^1^, 10^2^, 10^3^, and 10^4^ ng/mL and collagen peptide solution at concentrations of 0.25, 0.5, 1, and 2 × 10^3^ μg/mL. The control group was supplemented with an equivalent amount of DMEM basal medium. Following the incubation period, CCK-8 (M4839, AbMole, Houston, TX, USA) staining solution (1:10, 100 μL) was added to each well and incubated for two hours in darkness. The cell proliferation of PSC, market bovine Achilles tendon collagen (MPSC), SCP, and market fish scale collagen tripeptide (MSCP) were preliminarily assessed using a Cell Counting Kit-8 according to the manufacturer’s instructions.

### 3.12. Cell Migration Assay

The effect of collagen and collagen peptide on the migration of HFF-1 cells was assessed using an in vitro scratch test. HFF-1 cells in the logarithmic growth phase were seeded at a density of 1 × 10^6^ cells per well in six-well plates and cultured until reaching 100% confluence. A sterile pipette tip (200 µL) was then used to create a scratch on the bottom cell layer of each well. After washing with PBS, a serum-free medium (1.5 mL) containing optimal concentrations of PSC, MPSC (1 × 10^3^ ng/mL) and SCP, MSCP (1 × 10^3^ µg/mL) was added for cell culture. Cell migration was monitored under an inverted microscope every 12 h. Quantitative analysis of the scratch area was performed using Image J (Version:2.14.0/1.54f) and Photoshop software (Adobe photoshop 24.0.0), and the wound closure (%) was calculated according to the following formula.

$$Wound\;closure\left(\%\right)=\frac{Area\;between\;cells\;at\;0\;h-Area\;between\;cells\;at\;12\;h/24\;h}{Area\;between\;cells\;at\;0\;h}\times 100\%$$

### 3.13. Protein Expression Assay

Immunofluorescence staining (IF) and enzyme-linked immunosorbent assay (ELISA) were utilized to detect the expression of type I collagen (COL-I). HFF-1 cells were cultured in 6-well plates with a seeding density of 5 × 10^5^ cells per well and incubated for 24 h in a serum-free medium containing the same drug concentration described in Section 3.12. The expression of COL-I was subsequently detected using a universal IF toolbox. Briefly, cells were fixed with 2 mL of 4% paraformaldehyde for 30 min, washed three times with PBS, blocked by membrane rupture, and then incubated overnight at 4 °C with anti-type I collagen (1:200). The following day, goat anti-rabbit IgG DyLight™549 secondary antibody was added to the wells for one hour at room temperature in the dark. Cells were counterstained with DAPI in the dark for ten minutes at room temperature followed by CellMask™ Green plasma membrane staining. Inverted fluorescence microscopy was used to observe the fibroblast expression of COL-I and capture sample images of collagen peptide and the negative control. After culturing without staining for twenty-four hours, cells were lysed using RIPA lysate solution before being centrifuged at 3000 r/min for ten minutes to collect supernatant samples, which underwent ELISA analysis to determine COL-I content levels. Similarly, the expression of integrin α_2_β_1_, α_10_β_1_, and α_11_β_1_ was quantified using ELISA kits (MEIMIAN, Yancheng, China).

### 3.14. Damage Protection Analysis

An injury model was established in HFF-1 cells using H_2_O_2_ as an inducer. Briefly, cells were seeded in 96-well plates and cultured at the concentration indicated in Section 3.11, consistent with that of the original study. After 24 h of incubation, the medium was discarded. Subsequently, 100 μL of 900 μmol/L H_2_O_2_ was added to both the damage control and experimental wells and incubated for 2 h. The blank group did not undergo any treatment for inducing damage. Following injury induction, the same concentration of samples used prior to injury was added for further culture over 24 h, while the injury control group received basal medium without any drugs. Cell viability was assessed by measuring the OD value at 450 nm using a microplate reader. Simultaneously, the supernatant from each well was collected to determine the levels of inflammatory factors IL-2 and IL-6 using ELISA kits (MEIMIAN, Yancheng, China).

### 3.15. Statistical Analysis

The measured results were expressed as the mean ± standard deviation (SD) of triplicate experiments, and all experimental results were repeated three times. Statistical analysis was performed using GraphPad Prism 9 software, and the observed differences were found to be statistically significant (*p* < 0.05). Mapping was conducted using Origin software (2021 version, OriginLab Inc., Northampton, MA, USA).

## 4. Conclusions

In this study, we prepared squid skin collagen PSC and ASC, demonstrating their classification as type I collagen, and subsequently purified the 1.3 kDa squid collagen peptide SCP. The three-helix structure of both PSC and ASC was confirmed through UV, IR, and circular dichroism analyses; however, such confirmation was absent for SCP. LC-MS/MS analysis combined with de novo search identified 34 peptides present in type I collagen, revealing a repetitive sequence structure Gly-X-Y within the protein polypeptide chain. Collagen and its peptides exhibited favorable cell proliferation and migration properties while upregulating the expression of type I collagen; these cellular behaviors may be attributed to enhanced integrin receptor expression levels. Additionally, it is noteworthy that collagen and its peptides demonstrated anti-inflammatory and anti-oxidative effects while protecting fibroblasts against H_2_O_2_-induced damage. With excellent biocompatibility characteristics, there exists potential for utilizing collagen and its peptides as alternatives to standard mammalian collagens in medical applications. Further studies are necessary to incorporate these collagen peptides into a polymer hydrogel system for efficient drug delivery and tissue regenerative applications.

## Figures and Tables

**Table 1:** Amino acid composition of PSC, ASC, and SCP from squid skin (residues/1000 residues).

Amino Acid	PSC	ASC	SCP
Aspartic acid (Asp)	58.41 ± 0.56	59.41 ± 0.10	55.30 ± 0.03
Threonine (Thr)	25.92 ± 0.12	26.03 ± 0.03	29.04 ± 0.01
Serine (Ser)	34.64 ± 0.15	34.60 ± 0.05	41.69 ± 0.01
Glutamic acid (Glu)	84.58 ± 0.67	85.70 ± 0.10	95.58 ± 0.05
Glycine (Gly)	328.73 ± 1.22	335.10 ± 0.29	391.03 ± 0.22
Alanine (Ala)	89.38 ± 0.36	90.39 ± 0.08	116.85 ± 0.02
Valine (Val)	23.17 ±0.05	22.75 ± 0.03	25.09 ± 0.01
Methionine (Met)	1.72 ± 0.01	3.26 ± 0.01	0.00
Isoleucine (Ile)	15.40± 0.10	15.84 ± 0.02	16.94± 0.01
Leucine (Leu)	27.41 ±0.15	26.89 ± 0.03	32.62 ±0.01
Tyrosine (Tyr)	7.78 ± 0.11	5.63 ± 0.01	5.20 ± 0.01
Phenylalanine (Phe)	12.41 ± 0.12	11.05 ± 0.01	14.03 ± 0.01
Lysine (Lys)	13.28 ± 0.03	11.87 ± 0.01	12.00 ± 0.02
Histidine (His)	7.02 ±0.02	6.45 ± 0.01	9.14 ±0.01
Arginine (Arg)	56.88 ±0.25	57.29 ± 0.03	63.93 ± 0.01
Proline (Pro)	85.63 ±0.30	86.61 ±0.05	91.56 ± 0.02
Hydroxyproline (Hyp)	127.79 ± 0.58	121.13 ± 0.55	/
Total	1000.00	1000.00	1000.00

**Table 3:** Sequences of SCP polypeptides.

Source	Sequence	Mass	Score
Collagen type I alpha 1	GERGPKGPL	926.51	504.7
Collagen type I alpha 1	GLPGPQGPPGPS	1092.53	486.6
Collagen type I alpha 1	GPSGLPGI	729.38	444.5
Collagen type I alpha 1	GPPGLPGSA	752.39	442.4
Collagen type I alpha 1	SGPIGPA	598.32	434.4
Collagen type I alpha 1	GGPGFPGPQ	845.38	418.7
Collagen type I alpha 1	KGEQGHAGPAGPPGAPG	1516.71	416.9
Collagen type I alpha 1	NRGLPGPPGSQ	1095.55	415.9
Collagen type I alpha 1	PGPSGPR	667.35	412.5
Collagen type I alpha 1	GLPGPPGSQ	825.41	406.9
Collagen type I alpha 1	PGSPGPR	667.35	393.3
Collagen type I alpha 1	GERGLPGI	814.44	393.0
Collagen type I alpha 1	GPKGDDGQPGPPGFQ	1469.67	386.9
Collagen type I alpha 1	PGPAGPAG	655.310	385.9
Collagen type I alpha 1	GPKGDDGQPGPPGFQ	1485.66	370.8
Collagen type I alpha 1	GEAGPPGL	713.354	355.3
Collagen type I alpha 1	RSMLSQHM	989.47	347.6
Collagen type I alpha 1	PGSTGPA	586.28	346.0
Collagen type I alpha 1	VGPAGPA	568.31	341.5
Collagen type I alpha 1	GQPGPVGESGPIGPA	1335.65	339.8
Collagen type I alpha 1	GLPGIQ	600.34	338.3
Collagen type I alpha 1	GLPGPPGSQ	809.42	338.2
Collagen type I alpha 1	GPAGPR	554.31	337.0
Collagen type I alpha 1	SPGLQ	501.27	331.6
Collagen type I alpha 1	TGAPGFPGPRGP	1142.56	329.7
Collagen type I alpha 1	LPGIQ	543.31	329.3
Collagen type I alpha 1	RGFPGER	834.42	328.7
Collagen type I alpha 1	GLPGPQGPPGPS	1076.54	328.4
Collagen type I alpha 1	GPPGPS	527.25	320.0
Collagen type I alpha 1	PIGPKGEPGK	1011.55	309.5
Collagen type I alpha 1	GPAGSVGAP	712.36	302.6
Collagen type I alpha 2	GLPGLPGPT	824.45	467.3
Collagen type I alpha 2	GPPGLAGPS	768.39	389.7
Collagen type I alpha 2	PGLPGPT	654.35	300.7

## Data Availability

The data supporting the findings of this study are available upon reasonable request from the corresponding author.
